# Exciting inhibition in primates

**DOI:** 10.7554/eLife.59381

**Published:** 2020-07-01

**Authors:** Wim Vanduffel, Xiaolian Li

**Affiliations:** 1 Department of Neurosciences, KU Leuven Medical SchoolLeuvenBelgium; 2 Leuven Brain Institute, KU Leuven Medical SchoolLeuvenBelgium; 3 Department of Radiology, Harvard Medical SchoolBostonUnited States; 4 Martinos Center for Biomedical Imaging, Massachusetts General HospitalCharlestownUnited States

**Keywords:** optogenetics, vision, GABA, neurotransmitters, neurons, Rhesus macaque

## Abstract

A new genetic marker enables precise control over a group of inhibitory neurons in monkeys.

**Related research article** De A, El-Shamayleh Y, Horwitz GD. 2020. Fast and reversible neural inactivation in macaque cortex by optogenetic stimulation of GABAergic neurons. *eLife* **9**:e52658. doi: 10.7554/eLife.52658

At the age of 30, Louis Leborgne lost his ability to speak, and was only able to utter one syllable – ‘tan’. After his death 21 years later in 1861, a brain biopsy by his physician Paul Broca revealed that his inability to speak was caused by a lesion in the left frontal cortex, in a region of the brain now known as Broca’s area (Mohammed et al., 2018). More instructive though less known are Ernest Auburtin’s experiments on the exposed brain of a patient who had attempted suicide. When Auburtin softly pressed on specific parts of the frontal cortex, the patient lost his ability to speak, whereas his other cognitive functions remained unaffected (Stookey, 1963).

Whilst both studies showed the power of causally linking specific regions of the brain to specific behaviors, Auburtin’s experiments were more telling because of the reversible nature of his actions on an otherwise intact brain. However, these experiments – and many ‘causal’ techniques developed since then – lack spatio-temporal precision: that is, they cannot identify precisely where and when signals are produced or interrupted. Optogenetics overcomes these problems by using genetic manipulation or viral vectors to express light-sensitive proteins (such as opsins) in neurons: illumination with specific wavelengths of light can then induce neuronal responses that are precisely synchronized with the frequency of stimulation (Boyden et al., 2005). Moreover, specific cell types within a pool of diverse neurons can be precisely targeted, thereby achieving astonishing spatial resolution (Shemesh et al., 2017).

To reveal the causal relationship between a brain region and specific behavior, one can either increase local brain activity (Gerits et al., 2012), or decrease it (Afraz et al., 2015). Moreover, a region of the brain can be silenced by either inhibiting excitatory neurons or activating inhibitory neurons. A major drawback with the former approach, however, is that a period of silence induced by light is typically followed by a period of unwanted 'rebound' activity. Activating a local network of inhibitory neurons is more elegant because it takes advantage of natural inhibition in the brain and avoids the problem of rebound effects. This approach has proved successful in rodents, but efforts to translate it to primates have been limited. Recently, however, a new viral vector (Dlx5/6) that only targets inhibitory interneurons was demonstrated in multiple species, including primates (Dimidschstein et al., 2016).

Now, in eLife, Abhishek De (University of Washington), Yasmine El-Shamayleh (Columbia University) and Gregory Horwitz (University of Washington) report on the behavioral effectiveness of this approach in macaques (De et al., 2020). The researchers injected a Dlx5/6 vector carrying channelrhodopsin (an opsin that activates neurons) into the primary visual cortex of the primates and used immunohistochemical techniques to confirm that it primarily targeted inhibitory neurons that used the neurotransmitter GABA. When this region was illuminated via an optic fiber connected to an electrode, about two-thirds of the neurons increased their activity, whereas one third suppressed their activity. The latencies for the former group of neurons (that is, the interval between light stimulation and neuronal response) were short, which suggests that they were GABAergic interneurons that expressed channelrhodopsin. The latencies for the inactivated group of neurons were longer, indicating they were downstream from directly activated interneurons.

Therefore, using the new optogenetic technique to induce inhibition in the primary visual cortex (meaning this part of the cortex can no longer process visual information) should result in a temporary blind spot in the monkey’s visual field. To test this behaviorally, De, El-Shamayleh and Horwitz used a saccade test, during which they presented the monkey a point on a screen. When the position of the point overlapped with the receptive fields of the stimulated inhibitory neurons (blind spot), the monkeys were less likely to detect the stimulus (Figure 1). Also, performance on a contrast detection task was severely diminished when inhibitory neurons were activated. In this test, monkeys had to indicate (with eye movements) whether a contrast-adjusted faint stimulus was positioned either on the left or the right side of the screen. Optogenetic stimulation reduced their sensitivity to detect this cue on a grey background with the same mean luminance when the stimulus was placed inside the activated receptive fields. Thus, this experiment confirmed that optogenetics only affected the monkeys’ sensitivity to the visual stimulus, but not the ability to move their eyes.

**Figure 1.**
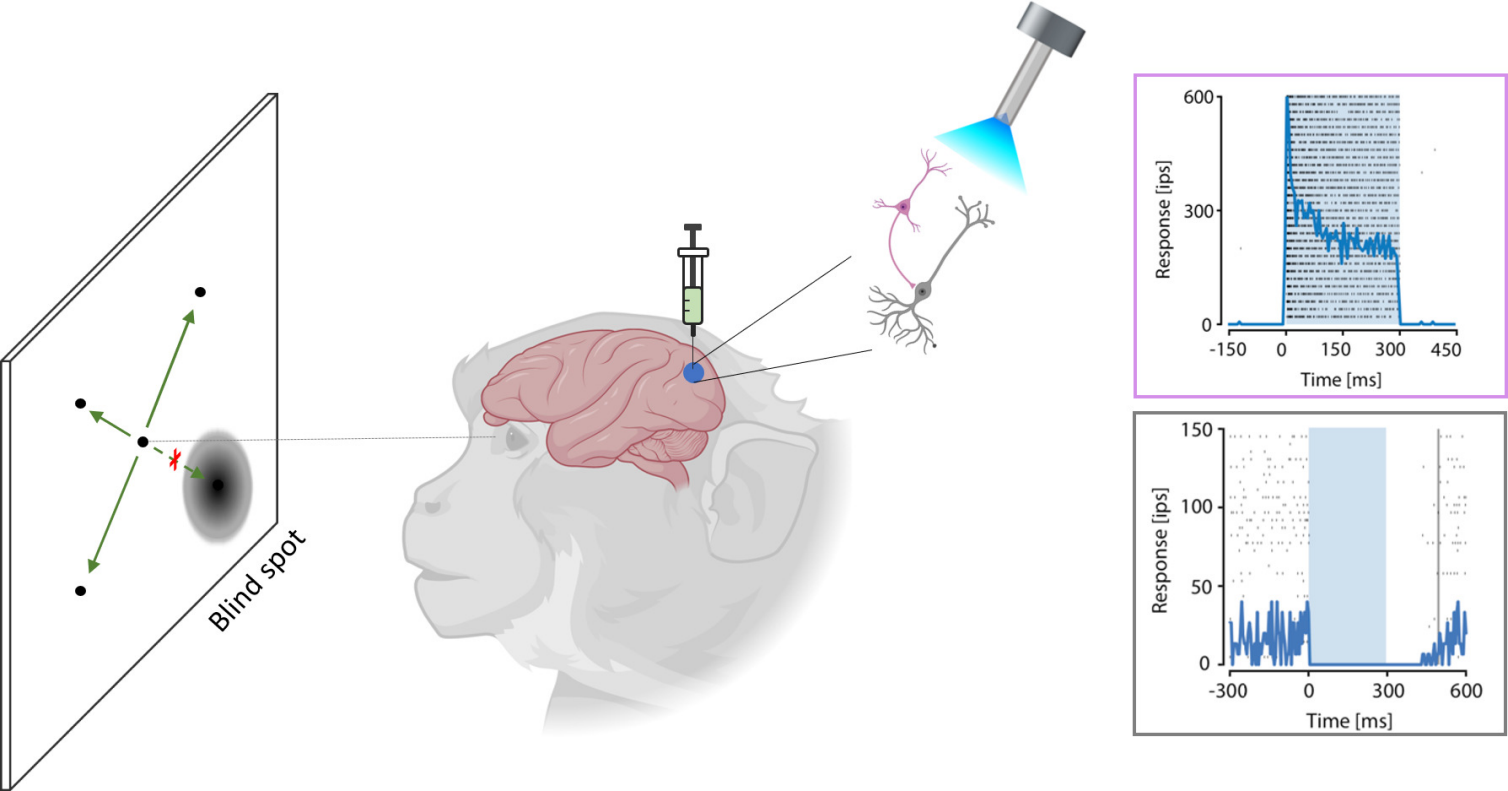
Local visual sensitivity is reduced in macaques by activating inhibitory neurons. A new optogenetics method can selectively stimulate inhibitory neurons and so reduce activity in a specific brain region. To test this method in macaques, De, El-Shamayleh and Horwitz injected the viral vector Dlx5/6 carrying channelrhodopsin – which activates inhibitory neurons (pink) – into the primary visual cortex of primates. Upon stimulation with light (torch), these inhibitory neurons were activated (top graph, blue line), subsequently leading to a deactivation of the excitatory neurons (grey: bottom graph, blue line). In a visual task, the monkeys were presented with a point that could appear randomly in different locations, and the subjects had to detect this point using an eye movement (green arrows). Activation of inhibitory neurons induced a blind spot in the visual field of the monkey; hence, monkeys were less likely to detect the point when it appeared in the blind spot (as indicated by the red cross). IPS: impulses per second (which corresponds to the firing rate of the neuron).

The work of De, El-Shamayleh and Horwitz constitutes another important milestone bridging the gap between research into the rodent brain and research into the monkey brain (Han et al., 2009; Gerits and Vanduffel, 2013), and it confirms that it is possible to successfully change behavior using selective activation of inhibitory neurons in primates (Dimidschstein et al., 2016). In the future, methods to reversibly control specific neurons in large brains with millisecond precision may become possible in monkeys (Marshel et al., 2019) and would allow to unravel the precise neuronal mechanisms underlying higher-order cognitive abilities unique to primates. Ultimately, it may be possible to recover lost brain functions in patients like Louis Leborgne, or patients with psychiatric disorders linked to a malfunctioning inhibitory circuitry of the brain.
